# Baicalin modulates NF-κB and NLRP3 inflammasome signaling in porcine aortic vascular endothelial cells Infected by *Haemophilus parasuis* Causing Glässer’s disease

**DOI:** 10.1038/s41598-018-19293-2

**Published:** 2018-01-16

**Authors:** Shulin Fu, Huashan Liu, Lei Xu, Yinsheng Qiu, Yu Liu, Zhongyuan Wu, Chun Ye, Yongqing Hou, Chien-An Andy Hu

**Affiliations:** 1 Hubei Key Laboratory of Animal Nutrition and Feed Science, Wuhan Polytechnic University, Wuhan, 430023 PR China; 2 Hubei Collaborative Innovation Center for Animal Nutrition and Feed Safety, Wuhan, 430023 PR China; 3 Biochemistry and Molecular Biology, University of New Mexico School of Medicine, Albuquerque, New Mexico, 87131 USA

## Abstract

*Haemophilus parasuis (H. parasuis)* can cause vascular inflammatory injury, but the molecular basis of this effect remains unclear. In this study,we investigated the effect of the anti-inflammatory, anti-microbial and anti-oxidant agent, baicalin, on the nuclear factor (NF)-κB and NLRP3 inflammasome signaling pathway in pig primary aortic vascular endothelial cells. Activation of the NF-κB and NLRP3 inflammasome signaling pathway was induced in *H. parasuis*-infected cells. However, baicalin reduced the production of reactive oxygen species, apoptosis, and activation of the NF-κB and NLRP3 inflammasome signaling pathway in infected cells. These results revealed that baicalin can inhibit *H. parasuis*-induced inflammatory responses in porcine aortic vascular endothelial cells, and may thus offer a novel strategy for controlling and treating *H. parasuis* infection. Furthermore, the results suggest that piglet primary aortic vascular endothelial cells may provide an experimental model for future studies of *H. parasuis* infection.

## Introduction

*Haemophilus parasuis* (*H. parasuis*) is a common commensal extracellular bacterium that colonizes the upper respiratory tract of swine and is responsible for causing Glässer’s disease, characterized by fibrinous polyserositis, polyarthritis, and meningitis^1^. The incidence of Glässer’s disease has increased as a result of changes in methods of swine feeding and management worldwide^2^. Fifteen serovars of *H*. *parauis* have been identified to date, but up to 25% of the isolates in some countries have not yet been serotyped^3,4^. A relationship has been observed between serovars and *H. parasuis* virulence^5^: Serovars 1, 5, 10, 12, 13 and 14 are considered to be highly virulent and cause high mortality in swine; serovars 2, 4, 8 and 15 show moderate virulence; while serovars 3, 6, 7, 9, and 11 are avirulent^6^. The existence of 15 serovars as well as non-typeable strains of *H. parasuis*, together with a lack of understanding of the pathogenesis of *H. parasuis* infection, means that the control of Glässer’s disease presents a challenge.

Proliferation of *H. parasuis* in the host cells can evoke a strong inflammatory immune response^7^, though the mechanisms responsible for the resulting in vascular inflammation and injury remain elusive. The nuclear factor-kappa B (NF-κB) signaling pathway is the predominant pathway induced by toll-like receptor (TLR) signaling, involving Iκκb-dependent phosphorylation of IκBa and/or IκBb, leading to their ubiquitination and degradation by the proteasome^8^. Pro-inflammatory cytokines and inflammatory responses play an important role in tissue injury and disease development, and are commonly regulated by NF-κB^9^. Previous research showed that the cell wall of *H. parasuis* contains lipopolysaccharide (LPS) molecules, which can elicit activation of NF-κB signaling pathways mediated by TLRS^10,11^. LPS can act via the TLR4 signaling pathway, subsequently triggering NF-κB activation and inflammatory cytokine production^12^. TLR4 also plays an important role in LPS-evoked tissue injury^12,13^. Importantly, some downstream targets of NF-κB, such as interleukin (IL)-6, IL-8, and tumor necrosis factor (TNF)-α, may in turn activate the NF-κB signaling pathway^14^. IL-6 is associated with acute and chronic inflammation^15^, while IL-8 elicits the recruitment and activation of neutrophils, which subsequently release reactive oxygen species (ROS) and cause local tissue injury and inflammation^11,16^. TNF-α has an important effect on both local and systemic inflammation^17^, and IL-10 and TNF-α have been shown to be involved in the adaptive response, which might contribute to protection against *H. parasuis* infection^18^. Pro-inflammatory cytokines may thus affect the inflammatory response induced by *H. parasuis*.

The immune system involves important protection mechanisms that defend against pathogens, such as bacteria and viruses. Innate immune cells, such as macrophages, can initiate inflammation, leading to the release of inflammatory cytokines during infection^19^. Inflammasomes are molecular platforms that elicit activation of caspase-1, resulting in the maturation of proinflammatory cytokines^20^. For example, NLRP3 inflammasomes are composed of NLRP3, apoptosis-associated speck-like protein including a CARD (N-terminal caspase recruitment domains) (ASC), and pro-caspase-1^21^. Excessive release of IL-1β has been reported to be involved in some systemic inflammatory diseases^22,23^, and was shown to cause local inflammation triggered by bacterial or viral infection, representing an important mechanism for fibrogenesis production^24,25^. Previous research demonstrated that NLRP3 inflammasomes in hepatic stellate cells were activated by *Schistosoma japonicum* infection, leading to the initiation of the inflammatory response and resulting in liver fibrosis^26^. Activation of the NLRP inflammasome also led to the stretch-induced inflammatory response in human periodontal ligament cells^27^. Although the innate adaptive immune response may protect animals from certain diseases, inappropriate activation of the NLRP3 inflammasome may contribute to disease progression and tissue injury, including neurodegenerative diseases^28,29^, metabolic diseases^30,31^, and sepsis^32,33^. *H. parasuis* has been shown to activate the NLRP3 inflammasome^34^, but the mechanism of vascular inflammation induced by *H. parasuis* remains unclear. We therefore determined if activation of the NLRP3 inflammasome triggered vascular inflammation leading to Glässer’s disease in porcine aortic vascular endothelial cells (PAVECs) infected with *H. parasuis*.

Baicalin (BA) is an effective plant-derived flavonoid and a traditionial Chinese medical herb isolated from *Scutellaria baicalensis* Georgi (Huang Qin). The chemical structure of BA has been determined^35^. BA possesses important functions, including anti-inflammatory^36–38^, anti-microbial^39,40^, and anti-oxidant activities^41,42^. Previous studies demonstrated that BA could reduce ROS production, suppress apoptosis, and inhibit the activation of the NF-κB and NLRP3 inflammasome signaling pathway in piglet mononuclear phagocytes treated with *H. parasuis*^34^. BA treatment also reduced T lymphocyte infiltration, gene expression of proinflammatory factors, and tissue damage in mice^37^. Furthermore, BA ameliorated LPS-induced inflammation and apoptosis in bovine mammary epithelial cells via inhibition of NF-κB activation and HSP72 upregulation^43^. BA also upregulated IRF4 protein expression and reversed LPS-induced macrophage subset redistribution, contributing to amelioration of inflammatory bowel diseases^44^. Moreover, BA significantly and selectively inhibited the viability of ovarian cancer cells, demonstrating its anti-cancer activity^45^. Overall, these results suggest that BA may inhibit the vascular inflammatory response induced by *H. parasuis*.

We previously showed that BA significantly inhibited the activation of NF-κB and the NLRP3 inflammasome during *H*. *parasuis* infection in piglet primary mononuclear phagocyte^34^. Importantly, some clinical phenotypes of Glässer’s disease, such as endocarditis and meningitis, are directly related to endothelial cells. However, the role of NF-κB and inflammasomes in porcine vascular endothelial cell injury has not been well-studied.

In the present study, we further explored the molecular mechanisms responsible for mediating the *H*. *parasuis*-induced activation of NF-κB and the NLRP3 inflammasome in porcine vascular endothelial cells. Our results revealed that BA could inhibit the *H. parasuis-*elicited inflammatory responses in PAVECs, and may thus represent a novel strategy for controlling and treating *H. parasuis* infection in pigs.

## Results

### Effect of baicalin on PAVECs viability *in vitro*

**Figure 1:**
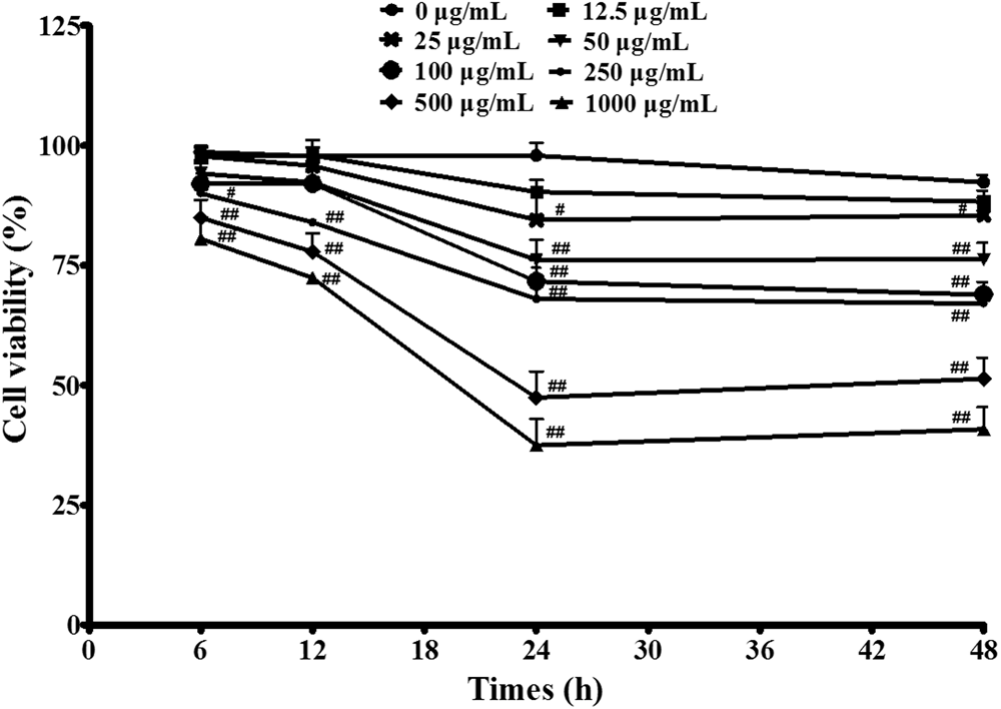
Effect of baicalin on PAVECs viability *in vitro*. Cells viability was measured by CCK-8 assay. The data was expressed as mean ± SD of triplicate samples form at least three independent experiments. ^#^P < 0.05 vs. control. ^##^P < 0.01 vs. control.

We determined the optimal concentration of BA by examining the viability of PAVECs at different concentrations. A decrease in the final concentration of BA from 250 μg/mL to 12.5 μg/mL for 12 h increased the cell viability from 83.9% to 97.7% (Fig. 1). BA demonstrated no significant cytotoxicity at concentrations of 12.5 μg/mL to 100 μg/mL for 12 h (*P* > 0.05) (Fig. 1). We also observed a dose-time relationship among BA concentration, stimulation time, and cell viability (Fig. 1). Based on these results, we used BA at 100 μg/mL for 12 h in subsequent experiments.

### Establishment of *H. parasuis* infection model using PAVECs

**Figure 2:**
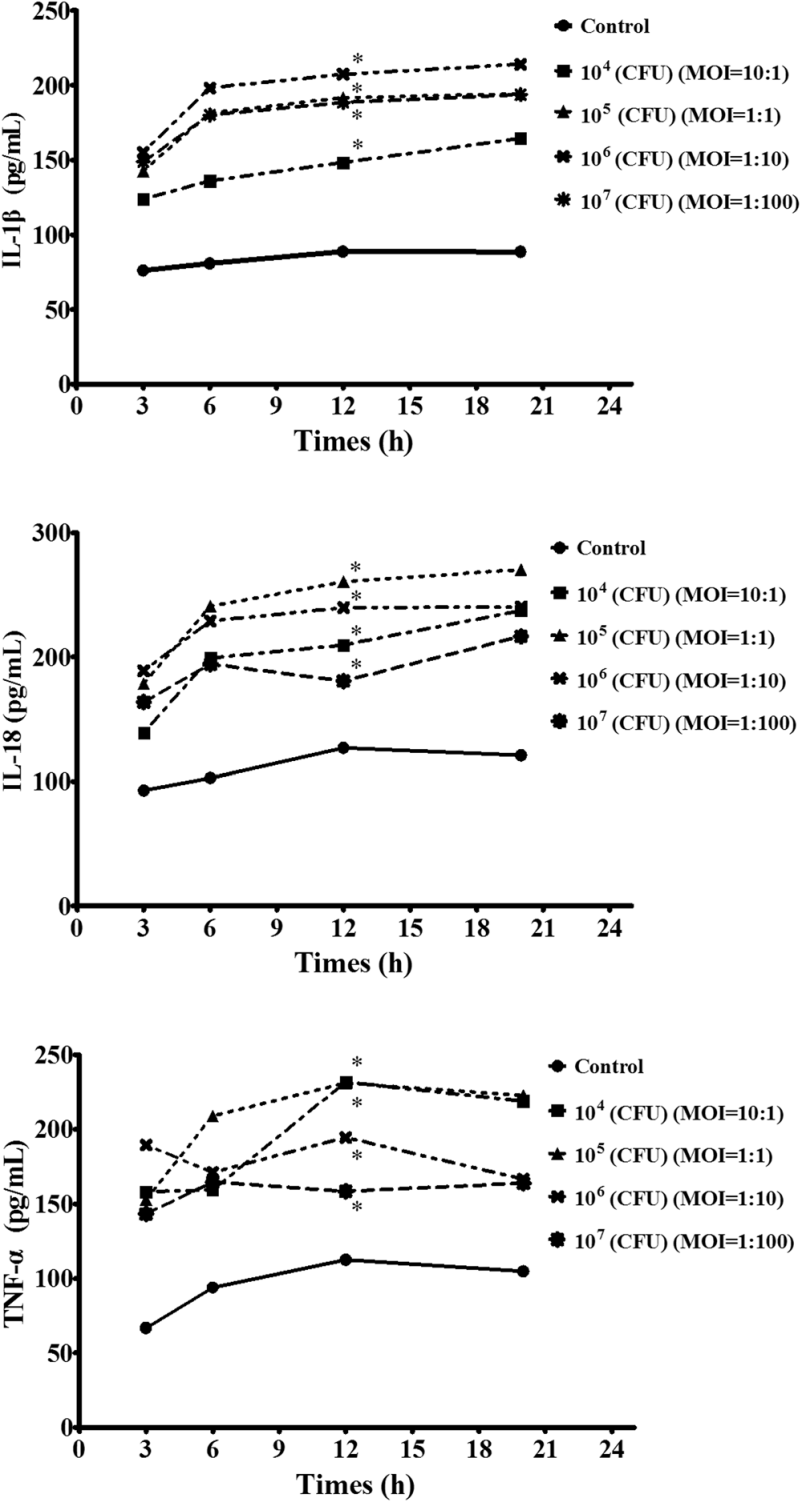
Establishment of *H. parasuis* infection model using PAVECs. The release of TNF-α, IL-1β and IL-18 was determined to explore the MOI and optimal interaction time. *Indicates significance at P < 0.05.

We explored the effect of the multiplicity of infection (MOI) of *H. parasuis* in the PAVEC infection model. Secretion of the inflammatory cytokines IL-1β, IL-18, and TNF-α into the supernatants by PAVECs tended to increase following *H. parasuis* infection at a MOI of 1:10 compared with negative control cells, but the difference was not significant (*P* > 0.05) (Fig. 2). However, secretion of these inflammatory cytokines after 12 h was significantly increased compared with the negative control group at MOIs of 1:1, 10:1, and 100:1 (*P* < 0.05) (Fig. 2). An MOI of 1:1 was therefore considered to represent the best infection model of the inflammatory response triggered by the *H. parasuis*.

### Effect of baicalin on production of proinflammatory cytokines triggered by *H. parasuis* in PAVECs

**Figure 3:**
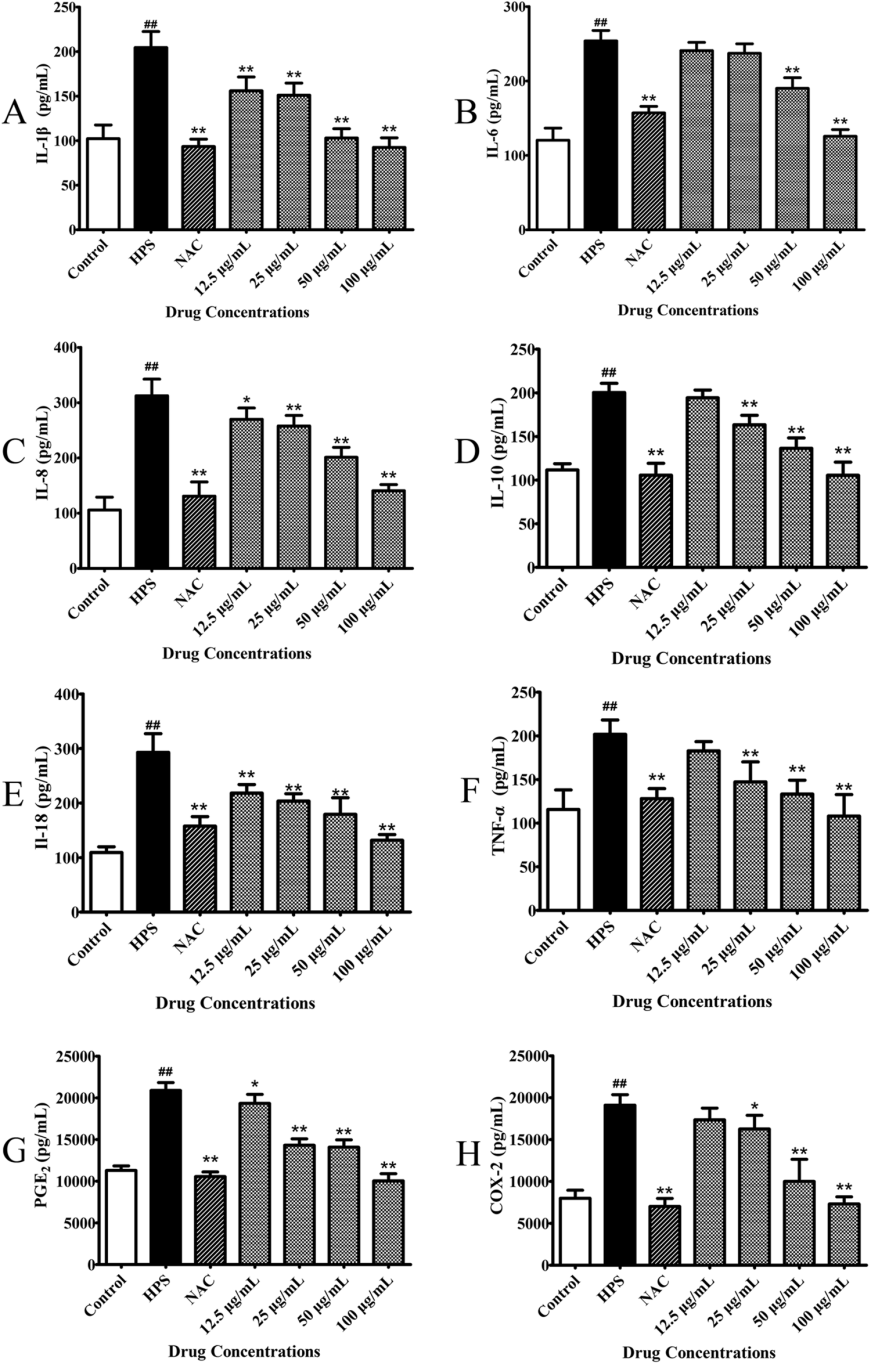
Effect of baicalin on release of proinflammatory cytokines triggered by *H. parasuis* in PAVECs. PAVECs were pre-treated with baicalin and co-cultured with *H. parasuis*. The release of proinflammatory cytokines in the cell culture supernatants was measured by ELISA assays. ^##^P < 0.01 vs. control. *Indicates significance at P < 0.05 and **indicates significance at P < 0.01.

We explored the release of proinflammatory cytokines from endothelial cells following *H. parasuis* infection. *H. parasuis* significantly induced the secretion of the proinflammatory cytokines IL-6, IL-8, IL-10, prostaglandin E_2_ (PGE2), cyclooxygenase (COX-2), IL-1β, IL-18, and TNF-α from PAVECs compared with the negative controls (*P* < 0.01), as measured by enzyme-linked immunosorbent assay (ELISA) (Fig. 3A–H). We also analyzed the induction of proinflammatory cytokines by NAC alone as a positive control, and found that NAC significantly inhibited the release of the above proinflammatory cytokines, compared with PAVECs infected with *H. parasuis* (*P* < 0.01) (Fig. 3A–H). Furthermore, pretreatment with BA at a final concentration at 50 or 100 μg/mL significantly decreased the production of the same proinflammatory cytokines in a dose-dependent manner compared with cells infected with *H. parasuis* alone (*P* < 0.01) (Fig. 3A–H).

**Figure 4:**
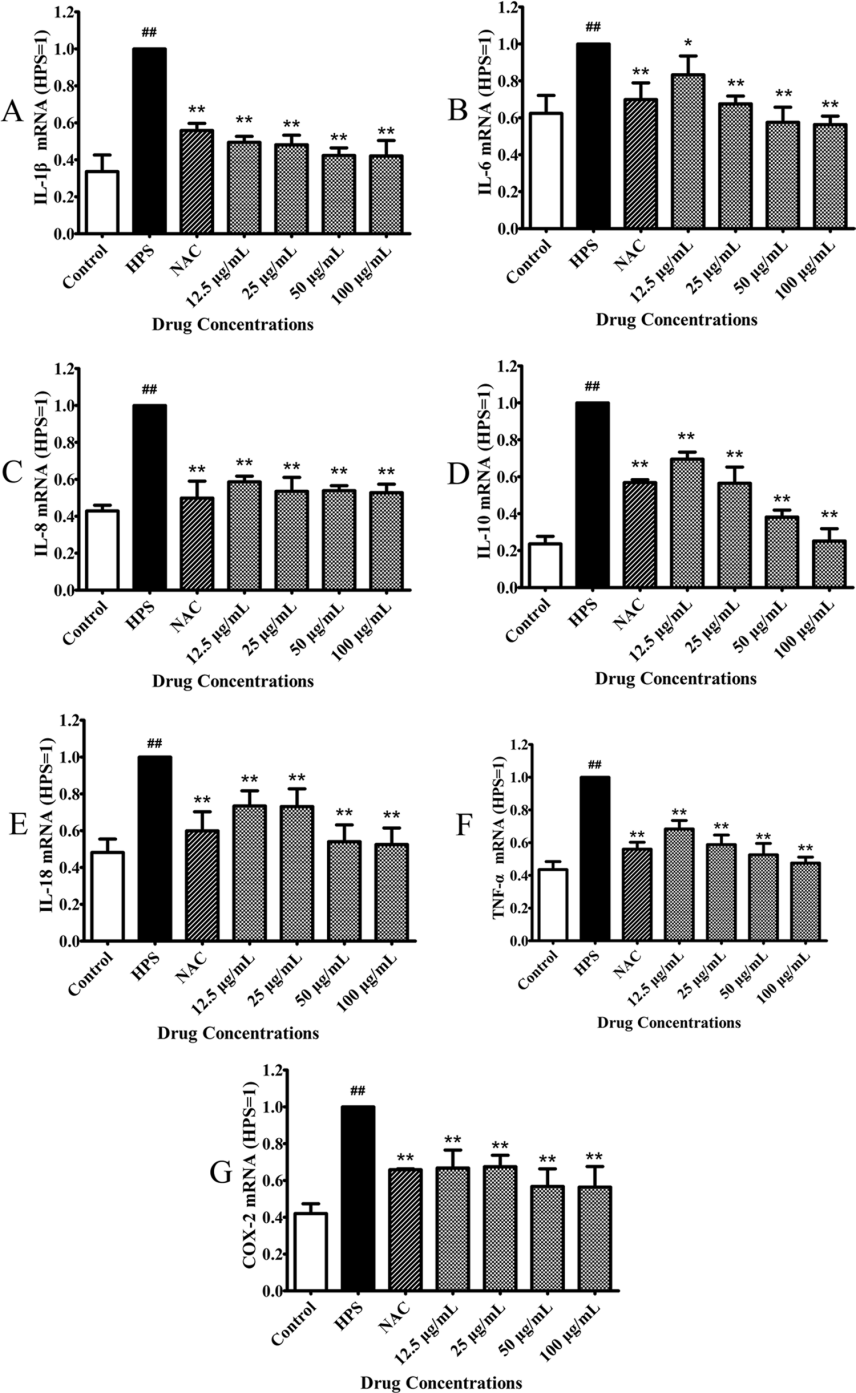
Effect of baicalin on proinflammatory cytokines expression triggered by *H. parasuis* in PAVECs. PAVECs were pre-treated with baicalin and incubated with *H. parasuis*. The expression of proinflammatory cytokines in the PAVECs were determined by qRT-PCR. ^##^P < 0.01 vs. control. *Indicates significance at P < 0.05 and **indicates significance at P < 0.01.

Furthermore, NAC significantly decreased the mRNA expression levels of IL-6, IL-8, IL-10, COX-2, IL-1β, IL-18, and TNF-α compared with cells infected with *H. parasuis* (*P* < 0.01), as determined by quantitative real-time-polymerase chain reaction (qRT-PCR) (Fig. 4A–G). mRNA expression levels of these pro-inflammatory cytokines were inhibited by pretreatment with BA (final concentration 12.5, 25, 50, or 100 μg/mL) in a dose-dependent manner compared with cells infected with *H. parasuis* (*P* < 0.05) (Fig. 4A–G).

### Effect of baicalin on *H. parasuis*-induced ROS release and cell apoptosis in PAVECs

**Figure 5:**
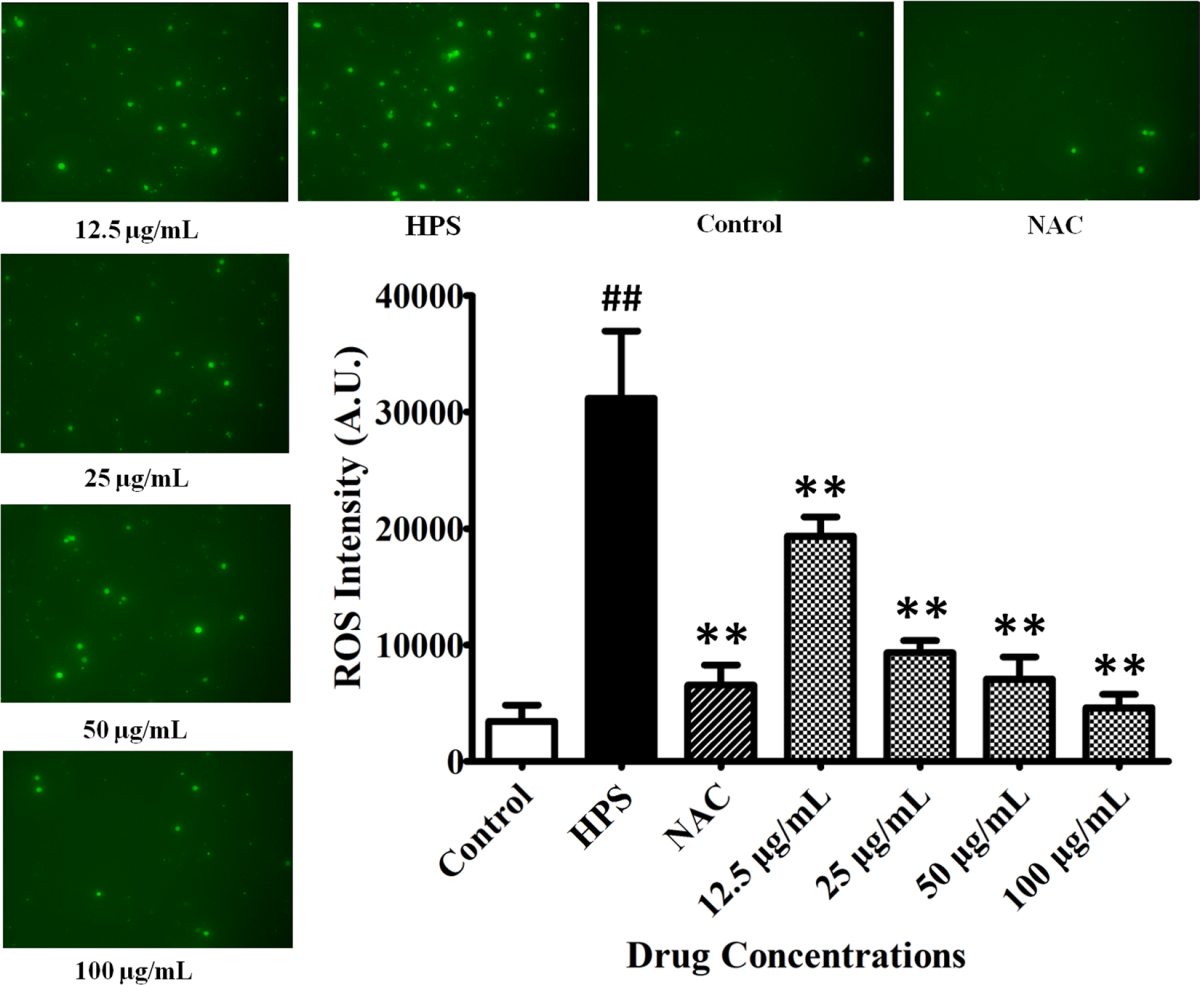
Effect of baicalin on *H. parasuis*-induced ROS release in PAVECS. PAVECs were stained with DCFH-DA and DHE and the fluorescence intensities were determined using the fluorescence microplate reader. ^##^P < 0.01 vs. control. **Indicates significance at P < 0.01.

We measured ROS production in PAVECs following stimulation with *H. parasuis* by measuring the fluorescence intensity. ROS production was markedly increased following *H. parasuis* infection for 12 h (*P* < 0.01) (Fig. 5), while BA treatment (final concentration 12.5–100 μg/mL) reduced ROS production (*P* < 0.01) (Fig. 5). Furthermore, NAC also markedly inhibited the generation of ROS in PAVECs compared with cells infected with *H. parasuis* (*P* < 0.01) (Fig. 5). Fluorescence microscopy analysis showed that the intracellular generation of ROS was decreased in a dose-dependent manner in both BA- and NAC-treated cells (Fig. 5).

**Figure 6:**
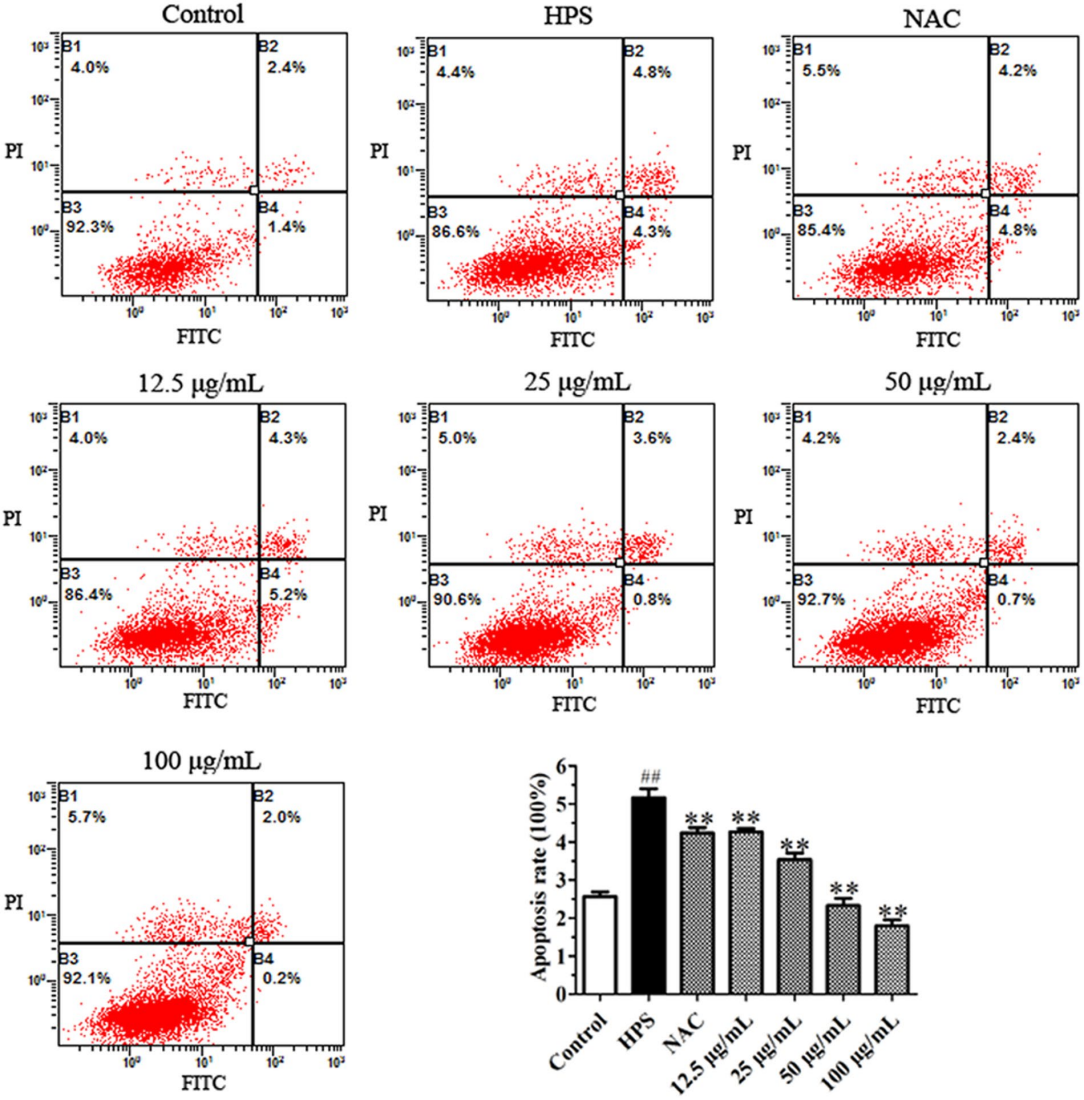
Effect of baicalin on *H. parasuis*-induced cell apoptosis in PAVECS. PAVECs were labeled with FITC Annexin V/PI and detected by flow cytometry. ^##^P < 0.01 vs. control. **Indicates significance at P < 0.01.

We also investigated the effect of *H. parasuis* on apoptosis in PAVECs, and showed that cells in the late stages of apoptosis were significantly increased following *H. parasuis* infection for 12 h compared with the negative control (*P* < 0.01) (Fig. 6). BA (final concentration 12.5–100 μg/mL) and NAC both significantly reduced the endothelial cells in the late stages of apoptosis triggered by *H. parasuis* (*P* < 0.01) (Fig. 6).

### Effects of baicalin on activation of the NF-κB signaling pathway triggered by *H. parasuis*

**Figure 7:**
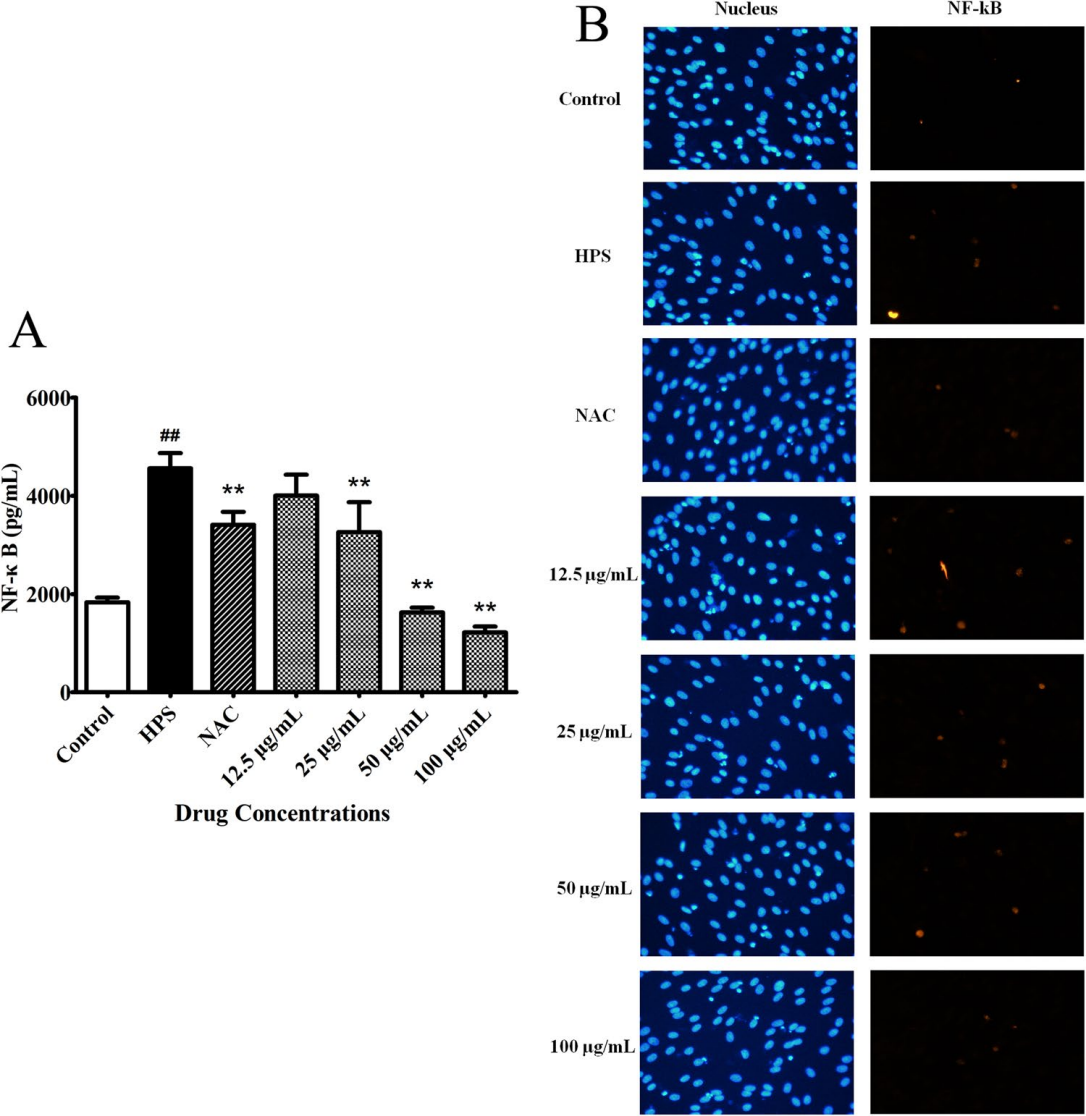
Effects of baicalin on activation of the NF-κB signaling pathway triggered by *H. parasuis*. (**A**) ELISA analysis of the levels of NF-κB p65 expression in the PAVECs. (**B**) Fluorescence observation of the levels of NF-κB p65 expression in the PAVECs. ^##^P < 0.01 vs. control. **Indicates significance at P < 0.01.

We investigated the effect of baicalin pretreatment on the NF-κB signaling pathway in PAVECs infected with *H. parasuis* by measuring levels of nuclear NF-κB p65 subunit by ELISA. Expression of nuclear NF-κB p65 subunit in PAVECs was markedly increased by *H. parasuis* infection for 12 h (*P* < 0.01) (Fig. 7A), but this increase was significantly inhibited by pretreatment with BA (final concentration of 25–100 μg/mL) compared with *H. parasuis* infection alone (Fig. 7A). We also detected the NF-κB p65 subunit by immunofluorescence microscopy and showed that p65 was upregulated in PAVECs by *H. parasuis* infection (Fig. 7B), and reduced again in cells pretreated with BA (final concentration 12.5–100 μg/mL) (Fig. 7B). NAC treatment (positive control) significantly reduced the p65 levels in endothelial cells (Fig. 7B).

### Effects of baicalin on activation of the NLRP3 inflammasome signaling pathway triggered by *H. parasuis*

**Figure 8:**
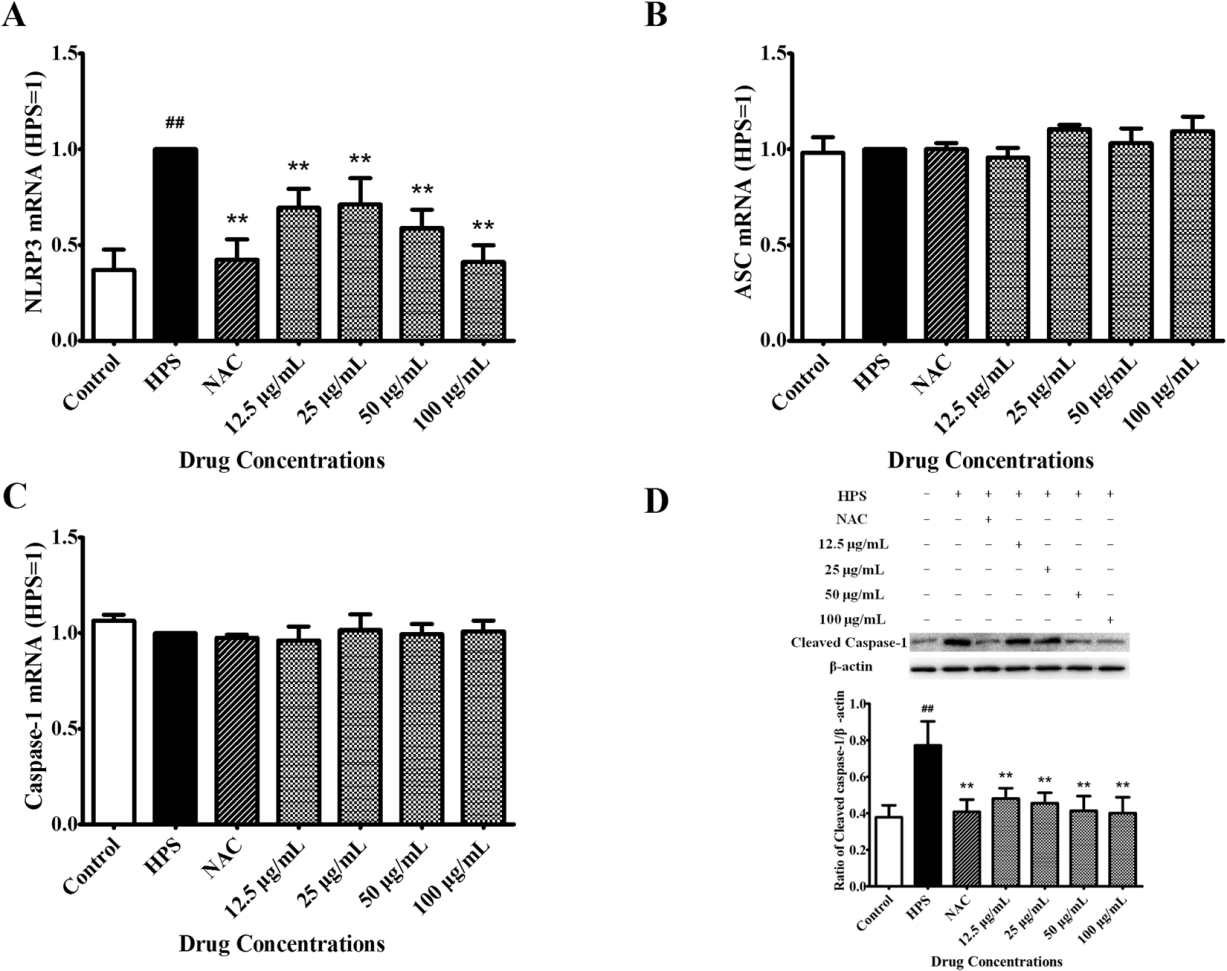
Effects of baicalin on activation of the NLRP3 inflammasome signaling pathway triggered by *H. parasuis*. (**A**,**B**,**C**) qRT-PCR analysis of the levels of NLRP3 inflammasome (NLRP3, ASC and Caspase-1) expression in the PAVECs. (**D**) Detection of the levels of cleaved caspase-1 in the PAVECs. ^##^P < 0.01 vs. control. **Indicates significance at P < 0.01.

We analyzed the effects of *H. parasuis* infection on the NLRP1, NLRP3 (NLRP3, ASC and caspase-1), NLRC4, and AIM2 inflammasomes in PAVECs by measuring the mRNA expression levels of NLRP1, NLRP3, NLRC4, and AIM2 by qRT-PCR. No NLRP1, NLRC4, or AIM2 mRNA expression was detected in PAVECs infected with *H. parasuis* for 12 h (data not shown), but NLRP3 mRNA levels were significantly up-regulated in PAVECs infected with *H. parasuis* for 12 h compared with the negative controls (*P* < 0.01) (Fig. 8A). NAC alone (positive control) significantly decreased the expression of NLRP3 at the mRNA level compared with *H. parasuis* infection alone (*P* < 0.01) (Fig. 8A). Pretreatment with BA (final concentration 12.5, 25, 50, or 100 μg/mL) significantly down-regulated NLRP3 mRNA expression compared with *H. parasuis* stimulation alone (*P* < 0.01) (Fig. 8A). However, *H. parasuis* did not induce ASC or caspase-1 mRNA expression in PAVECs (*P* > 0.05) (Fig. 8B,C). NAC and BA alone (12.5–100 μg/mL) also failed to affect mRNA expression levels of ASC and caspase-1 in PAVECs (*P* > 0.05) (Fig. 8B,C).

We also examined the expression levels of cleaved (active) caspase-1 protein in PAVECs following *H. parasuis* infection for 12 h by western blot. Activated caspase-1 protein expression was significantly up-regulated by *H. parasuis* compared with negative control cells, while NAC (positive control) significantly inhibited activated caspase-1 expression compared with *H. parasuis* infection (*P* < 0.01) (Fig. 8D). Pretreatment with BA (final concentration 12.5–100 μg/mL) significantly down-regulated activated caspase-1 protein expression compared with *H. parasuis* stimulation alone, in accord with the results for mRNA expression levels (*P* < 0.01) (Fig. 8D).

## Discussion

To our best of our knowledge, the current study provides the first evidence for the anti-inflammatory functions of baicalin in *H. parasuis*-infected PAVECs via inhibiting activation of the NLRP3 inflammasome and NF-κB signaling pathway, and thereby protecting the cells from *H. parasuis*-evoked inflammation.

Endothelial cells have previously been shown to play an active role during the inflammatory immune response to bacteria and their products^46^. Other studies also demonstrated a central role for endothelial cells in regulating inflammation^47,48^. In addition, endothelial cell activation is necessary for leukocyte recruitment to reduce the sepsis-associated increase in vascular permeability^49^. We therefore developed a suitable cellular model for evaluating endothelial cell activation during bacterial infection, and for improving our understanding of and developing ways of modulating inflammation. *H. parasuis* was previously shown to participate in serum resistance, adhesion, and invasion in studies using porcine kidney epithelial cells (PK-15)^50^, porcine aortic endothelial cells (AOC-45)^51^, porcine umbilical vein endothelial cells^52^, and newborn pig tracheal cells^53^. *H. parasuis* infection was shown to contribute to activation of the p38/JNK/mitogen-activated protein kinase pathway predominantly linked to inflammation in PK-15 cells^54^. In the current study, we used primary endothelial cells isolated from porcine aortas. To the best of our knowledge, this is the first report of the use of primary aortic vascular endothelial cells to study *H. parasuis* infection, and we suggest that this may provide a better system for exploring the inflammatory mechanisms of vascular injury during infection with *H. parasuis* and other bacteria. We previously established a model using peripheral blood monocytes^34^. The use of an infection model utilizing both types of primary cells (PAVECs and peripheral blood monocytes) could help to clarify the mechanism responsible for the vascular inflammation caused by *H. parasuis*.

In the present study, the secretion of IL-6, IL-8, and TNF-α by endothelial cells was significantly increased by *H. parasuis*, consistent with the previous finding that *H. parasuis* stimulated the release of IL-6 and IL-8 by newborn pig tracheal cells^53^. TNF-α has the characteristics of a multifunctional pro-inflammatory cytokine, with an important role in the pathogenesis of inflammatory diseases^9^. IκB kinase, which is activated by TNF-α, has been reported to phosphorylate IκB and induce its degradation, leading to the liberation of NF-κB, and evoking expression of a variety of genes that participate in inflammatory responses, such as IL-6 and IL-8^55^. Notably, overexpression of IL-8 has been related to some lung diseases^56^. In addition, IL-10 induced humoral immunity to kill extracellular microbial pathogens and produce protective antibodies^57,58^. *H. parasuis* was shown to activate the inflammatory transcription factor NF-κB in a time- and dose-dependent manner in PK-15 cells, through IκB degradation and release of IL-8 and CCL4^11,54^. Overexpression of porcine Coro1A down-regulated NF-κB and thereby inhibited the transcription of the NF-κB-mediated downstream genes IL-6, IL-8, and COX-2^59^. Our results indicated that *H. parasuis* could activate the NF-κB signaling pathway in aorta endothelial cells. Furthermore, this activation was significantly inhibited by baicalin, suggesting that baicalin exerted important effects resulting in reduced *H. parasuis*-evoked inflammation.

Inflammasomes are intracellular protein complexes that play an important role in innate immune sensing^60^. Previous research showed that *Streptococcus pneumoniae* could induce NLRP3-dependent IL-1β production, related to a pro-inflammatory cytokine cascade^61^. In addition, the degree of caspase-1 activation via the NLRP3 inflammasome was associated with clinical disease severity in patients with *S. pneumoniae* infection^62^. *Staphylococcus aureus* α-hemolysin was linked to an IL-1β response, which evoked NLRP3-dependent activation of caspase-1 in mouse and human monocytic cells^63,64^. To determine if activation of the NLRP3 inflammasome occurred in aorta endothelial cells stimulated with *H. parasuis*, and to explore the molecular mechanisms mediating inflammasome activation, we established an *H. parasuis* cell-infection model. We demonstrated that NLRP3, ASC, and caspase-1 aggregation, and IL-1β expression, were significantly increased in endothelial cells infected with *H. parasuis*. However, activation of the NLRP3 inflammasome was markedly attenuated in cells pretreated with baicalin. We therefore inferred that activation of the inflammasome was accompanied by the development of vascular inflammation, ultimately leading to Glässer’s disease as a result of overexpression of proinflammatory cytokines or chemokines during *H. parasuis* infection. This suggests that the development of vascular inflammation might be related to activation of caspase-1 via inflammasomes, though further studies are needed to clarify the molecular mechanisms responsible for the development of local inflammation.

Previous research demonstrated that ROS released by NADPH oxidase may act as defense and signaling molecules linked to innate immunity and various kinds of cellular responses^65,66^. An imbalance between ROS and antioxidant enzymes could lead to cytotoxicity, thus contributing to the pathogenesis of chronic diseases^67^. In this study, we used NAC as a positive control, and showed that both NAC and baicalin could significantly inhibit the production of ROS. NAC has been considered as a ROS-specific inhibitor. SEA-induced NLRP3 inflammasome formation and activation in hepatic stellate cells were significantly attenuated or abolished by NAC^26^. Inhibitors of ROS release and ROS scavengers have been reported to inhibit activation of the NLRP3 inflammasome^68^; however, ROS inhibition could not affect activation of the NLRP3 inflammasome directly, but could negatively regulate the priming step of inflammasome activation^69^. We therefore speculated that excessive ROS production may activate the relevant inflammatory signaling pathway leading to the vascular inflammatory response, which could be inhibited by baicalin. Further studies are needed to determine the specific mechanisms involved.

Overall, the results of the current study demonstrate that *H. parasuis* activates the NLRP3 inflammasome and NF-κB signaling pathway in PAVECs, while these effects can be significantly inhibited by baicalin. We aim to conduct further studies to validate the anti-inflammatory effect of baicalin in a pig model of *H. parasuis* infection *in vivo*.

## Methods

### Bacterial strain, growth conditions and drug

*H. parasuis* SH0165 strain, isolated from the lung of a commercial pig with arthritis, fibrinous polyserositis, hemorrhagic pneumonia, and meningitis, is a highly virulent strain of serovar 5^70,71^. The SH0165 strain was grown in tryptic soy broth (Difco Laboratories, USA) or tryptic soy agar (Difco Laboratories) supplemented with 10 μg/ml of NAD (Sigma, USA) and 10% newborn calf serum (Gibco, USA) under 37 °C.

Baicalin was obtained from the National Institutes for Food and Drug Control (Beijing, China; B110715–201318) and dissolved and diluted in RPMI-1640 medium (Gibco, USA).

### Isolation and culture of PAVECs

This study was carried out in strict accordance with the recommendations of the China Regulations for the Administration of Affairs Concerning Experimental Animals 1988 and Hubei Regulations for the Administration of Affairs Concerning Experimental Animals 2005. The protocol was approved by China Hubei Province Science and Technology Department (permit number SYXK(ER) 2010-0029). All experimental animals were euthanized at the end of the experiments.

Three 35-day-old naturally farrowed, early-weaned piglets (Duroc × Landrace × large white) weighing 7–10 kg, validated to be negative for antibody against *H. parasuis* by INGEZIM Haemophilus 11. *H. parasuis*. K1 (INGEZIM, Spain), were obtained from Wuhan COFCO Meat Product Co., Ltd. (Wuhan, China) and used for *in vitro* experiments.

PAVECs were isolated and identified as described previously^72^. Briefly, the uptake of Ac-LDL was determined by incubating PAVECs with 10 μg/ml of 1,1′-dioctadecyl-3,3,3′,3′-tetramethylindocarbocyanine (DiI)-labelled Ac-LDL (Invitrogen, USA) in cell medium for 12 h at 37 °C. Then the cells were washed three times with PBS, detached by trypsinization and analyzed by fluorescence microscopy (Fig. S1). The PAVECs were cultured as described previously, with some minor modifications^73^. Briefly, endothelial cells were obtained in small sheets after treatment of the aorta lumen (20 min, 37 °C) with 0.1% type I collagenase (Sigma, USA) in M-199 medium (Gibco, USA) containing penicillin-streptomycin solution (Gibco, USA). The suspension was centrifuged at 100 × g for 10 min, and the cells from one aorta were resuspended in 5 mL of M-199 containing 20% fetal bovine serum (FBS) (Gibco, USA), and then plated in a T-25 tissue-culture plate (Costar, USA). Endothelial cells were counted and their viability was determined by Trypan blue exclusion.

### Effect of dosing schedule on PAVECs viability *in vitro*

The viability of PAVECs was measured by Cell Counting Kit-8 (CCK-8) assay (Dojindo Molecular Technologies, Japan)^74^. Briefly, PAVECs were seeded into 96-well plates (Costar, USA) at 1 × 10^5^ cells/well and then treated with baicalin at a final concentration of 0, 12.5, 25, 50, 100, 250, 500, or 1000 μg/mL for 6, 12, 24, or 48 h at 37 °C under 5% CO_2_. CCK-8 solution (10 μL) was added to each well and incubated for 90 min at 37 °C, and the optical density was then measured at 450 nm. Cell viability was calculated according to the following formula: cell viability (%) = (experimental well − blank well/ control well − blank well) × 100%. The data were expressed as mean ± standard deviation of triplicate samples from at least three independent experiments.

### PAVEC model of *H. parasuis* infection

To confirm the MOI of *H. parasuis* in the endothelial cells, 1 × 10^5^ cells were seeded into 96-well plates followed by the addition of *H. parasuis* at 10^4^, 10^5^, 10^6^, or 10^7^ CFU/mL and co-culture at 37 °C under 5% CO_2_ for 3, 6, 12, and 20 h, respectively. Release of the inflammatory cytokines TNF-α, IL-1β, and IL-18 from the cell culture supernatant was determined to explore the MOI and optimal interaction time.

### Measurement of ROS and cell apoptosis

Intracellular ROS were detected by DCFH-DA staining^75^. Briefly, 2 × 10^5^ PAVECs were seeded into 24-well plates (Costar) and treated with baicalin at final concentrations of 12.5, 25, 50, and 100 μg/mL, respectively, for 1 h. *H. parasuis* 2 × 10^5^ CFU/mL was added into the plates and co-cultured for 12 h. After 12 h, the incubations were washed three times with sterile phosphate-buffered saline and stained with 10 μM DCFH-DA and 5 μM DHE (Nanjing Jiancheng Bioengineering Institute, Nanjing, China) at 37 °C under 5% CO_2_ for 30 min. The fluorescence intensities were then determined using a fluorescence microplate reader (Olympus, Japan). Apoptosis was detected using FITC Annexin V Apoptosis Detection Kit I (BD Pharmingen, USA) according to standard procedures, and examined by flow cytometry^76^. Briefly, 2 × 10^5^ PAVECs were seeded into 24-well plates (Costar) and then treated with baicalin at a final concentration of 12.5, 25, 50, or 100 μg/mL, respectively, for 1 h. *H. parasuis* at 2 × 10^5^ CFU/mL was then added into the plates and co-cultured for 12 h. The incubations were then washed three times with sterile phosphate-buffered saline and stained with FITC Annexin V and Propidium Iodide (PI). Cell apoptosis was measured by flow cytometry (Beckman Coulter, FC500, USA).

### Determination of proinflammatory cytokine concentrations

Levels of the inflammatory cytokines IL-1β, IL-18, TNF-α, IL-6, IL-8, IL-10, PGE2, and COX-2 were measured in the cell culture supernatants by ELISA assays (R&D, USA), according to the manufacturers instructions. Briefly, 1 × 10^5^ PAVECs were seeded into 24-well plates and pre-treated with baicalin at a final concentration of 12.5, 25, 50, or 100 μg/mL for 1 h. *H*. *parasuis* 1 × 10^5^ CFU/mL were then added into the wells and co-cultured for 12 h. The cell supernatants were collected and centrifuged for 20 min at 400 × *g* at 4 °C, and the levels of inflammatory cytokines were measured by ELISA assays.

### Total RNA extraction and qRT-PCR determination

We also measured the gene expression levels of IL-1β, IL-18, TNF-α, IL-6, IL-8, and IL-10, as well as the NLRP1, NLRP3 (NLRP3, ASC, and caspase-1), NLRC4, and AIM2 inflammasomes in PAVECs infected with *H. parasuis*. PAVECs at 1 × 10^7^ were seeded into 24-well plates and incubated with baicalin at a final concentration of 12.5, 25, 50, or 100 μg/mL for 1 h, followed by the addition of 1 × 10^7^ CFU/mL *H. parasuis* for 12 h. After co-culture, the PAVECs were collected and total cellular RNA was extracted using TRIzol reagent (Invitrogen, USA). The RNA was reverse-transcribed to cDNA using reverse transcriptase (TaKaRa, Dalian, China) and cDNA was amplified and measured using a SYBR Green PCR Kit (TaKaRa, Dalian, China) according to the manufacturer’s instructions. Individual transcripts in each sample were repeated at least three times and β-actin was used as the internal control. The nucleotide sequences of the primers used for qPCR are listed in Table 1.

**Table 1:** Primers for qRT-PCR.

Gene	Nucleotide sequence (5′-3′)	Tm (°C)	Length (bp)
β-actin	Forward TGCGGGACATCAAGGAGAAG	57.4	216
	Reverse AGTTGAAGGTGGTCTCGTGG	57.4	
NLRP3	Forward GGAGGAGGAGGAAGAGGAGATA	59.5	147
	Reverse AGGACTGAGAAGATGCCACTAC	57.7	
ASC	Forward ACAACAAACCAGCACTGCAC	55.4	126
	Reverse CTGCCTGGTACTGCTCTTCC	59.5	
Caspase-1	Forward GAAGGAGAAGAGGAGGCTGTT	57.6	268
	Reverse AGATTGTGAACCTGTGGAGAGT	55.8	
IL-1β	Forward TCTGCATGAGCTTTGTGCAAG	55.6	225
	Reverse ACAGGGCAGACTCGAATTCAAC	57.7	
IL-18	Forward AGTAACCATCTCTGTGCAGTGT	55.8	155
	Reverse TCTTATCATCATGTCCAGGAAC	53.9	
TNF-α	Forward CGCTCTTCTGCCTACTGCACTTC	61.3	164
	Reverse CTGTCCCTCGGCTTTGACATT	57.6	
IL-6	Forward CCAGGAACCCAGCTATGAAC	57.4	142
	Reverse CTGCACAGCCTCGACATT	54.9	
IL-8	Forward CAGAGCCAGGAAGAGACT	54.9	461
	Reverse GACCAGCACAGGAATGAG	54.9	
IL-10	Forward GCATCCACTTCCAGGCCA	57.2	176
	Reverse CTTCCTCATCTTCATCGTCA	53.4	
COX-2	Forward CTGTCCCATCCCTCGGTTTA	54.4	105
	Reverse TCTCTGAGCACTGTCCGTAAT	54.4	
NLRP1	Forward AGAACCTCGCATAGTCATCA	50.1	276
	Reverse CATCCTGGCTCATCTACAC	50.2	
NLRC4	Forward TTCTCCTTGATGGCTACAGTGA	54.9	109
	Reverse TGTGGTGGCAGTAACATTGAC	54.7	
AIM2	Forward GTAGTCCAGAAGGTAACAGAA	50.2	193
	Reverse TGCTATGAACTCCAGATGTC	50.2	

### Detection of NF-κB p65 nuclear translocation by ELISA and immunofluorescence

We determined the effects of baicalin pretreatment on NF-κB signaling in PAVECs infected with *H. parasuis* by measuring p65 levels in monocytes. PAVECs at 1 × 10^7^ were seeded into 6-well tissue culture plates and pretreated with NAC (1 mM) or baicalin (12.5, 25, 50, 100 μg/mL), respectively, for 1 h. *H. parasuis* at 1.0 × 10^7^ CFU/mL was then added and co-cultured for 12 h at 37 °C under 5% CO_2_. The cells were then collected and cytoplasmic and nuclear proteins were extracted using a cytosolic–nuclear protein extraction kit (Beyotime Biotechnology, Shanghai, China). Protein concentrations were measured using bicinchoninic acid protein assay reagents (Beyotime Biotechnology), according to the manufacturer’s instructions. NF-κB p65 protein levels in the cytoplasm and nucleus were determined using an NF-κB ELISA kit (Blue Gene Biotechnology, Shanghai, China), and nuclear translocation of NF-κB p65 was expressed as the ratio of nuclear/cytoplasmic expression of NF-κB p65 protein.

We also investigated NF-κB p65 nuclear translocation by immunofluorescence^77^. Briefly, 1 × 10^6^ PAVECs were seeded into 6-well plates with cover slips. The cells were pretreated with NAC (1 mM) or baicalin (12.5, 25, 50, 100 μg/mL), respectively, for 1 h and then co-cultured with 1 × 10^6^ CFU/mL *H. parasuis* for 12 h, followed by fixing with 4% paraformaldehyde for 1 h and permeabilization with 0.5% Triton X-100 for 30 min. The cells were blocked with goat serum for 1 h, and incubated with anti-NF-κB p65 antibody (NF-κB Activation, Nuclear Translocation Assay Kit, Beyotime) at 4 °C overnight. After washing three times, the slices were further incubated with anti-rabbit Cy3 antibody (Beyotime) for 1 h at 37 °C and counterstained with 4’,6-diamidino-2-phenylindole. The slices were then visualized and images were captured with a fluorescence microscope.

### Western blotting

PAVECs at 1 × 10^7^ CFU/mL were pretreated with NAC (1 mM) or baicalin (final concentration 12.5, 25, 50, or 100 μg/mL) for 1 h, respectively, and *H. parasuis* at 1.0 × 10^7^ CFU/mL was then added into the plate wells and co-incubated for 12 h. The cells were collected and total cell protein was extracted using a total protein extraction kit (Beyotime Biotechnology). The protein concentration was determined using a bicinchoninic acid protein assay kit (Sigma). Total cell proteins were isolated by 12% sodium dodecyl sulfate-polyacrylamide gel electrophoresis and transferred onto a polyvinylidene difluoride membrane and blocked with 5% skim milk for 1 h at 25 °C. After washing five times in TBST, the membrane was was cultured with cleaved caspase-1 antibody or β-actin antibody (Cell Signaling Technology, USA) for 12 h at 4 °C, followed by five washes in TBST, incubation with horseradish peroxidase-linked goat anti-rabbit antibody (Proteintech, USA) at 25 °C for 1 h, and then visualized using ECL solution (Thermo Pierce ECL, USA). The expression levels of cleaved caspase-1 and β-actin were measured using a FluorChem FC2 AIC system (Alpha Innotech, USA).

### Statistical analysis

The experimental data were expressed as mean ± SD. The difference between two groups was analyzed using the two-tailed Student *t* test. *P* values of <0.05 were considered significant. *p < 0.05; **p < 0.01 and ***p < 0.001.

## Electronic supplementary material

Supplementary Information
