# Supplementary material for: Baicalin modulates NF-κB and NLRP3 inflammasome signaling in porcine aortic vascular endothelial cells Infected by Haemophilus parasuis Causing Glässer’s disease
Source: Sci Rep. 2018 Jan 16;8:807. doi: 10.1038/s41598-018-19293-2 (PMC5770393; doi:10.1038/s41598-018-19293-2)
Supplement: Supplementary file 1 — Supplementary Information [file 41598_2018_19293_MOESM1_ESM.doc]

**Baicalin modulates NF-κB and NLRP3 inflammasome signaling in porcine aortic vascular endothelial cells Infected by *Haemophilus parasuis* Causing Glässer's disease**

Shulin Fu ^1, 2†^, Huashan Liu ^1†^, Lei Xu ^1†^, Yinsheng Qiu ^1, 2^,*, Yu Liu ^1, 2^, Zhongyuan Wu ^1, 2^, Chun Ye ^1, 2^, Yongqing Hou ^1, 2^, Chien-An Andy Hu ^1, 3^

*^1^ Hubei Key Laboratory of Animal Nutrition and Feed Science, Wuhan Polytechnic University, Wuhan 430023, PR China*

*^2^ Hubei Collaborative Innovation Center for Animal Nutrition and Feed Safety, Wuhan 430023, PR China*

*3 Biochemistry and Molecular Biology, University of New Mexico School of Medicine, Albuquerque, New Mexico 87131, USA*

*^†^* These authors contributed equally to the work.

* Corresponding author:

Yinsheng Qiu, Hubei Key Laboratory of Animal Nutrition and Feed Science, Wuhan Polytechnic University, Wuhan 430023, PR China.

E-mail address: qiuyinsheng6405@aliyun.com

**
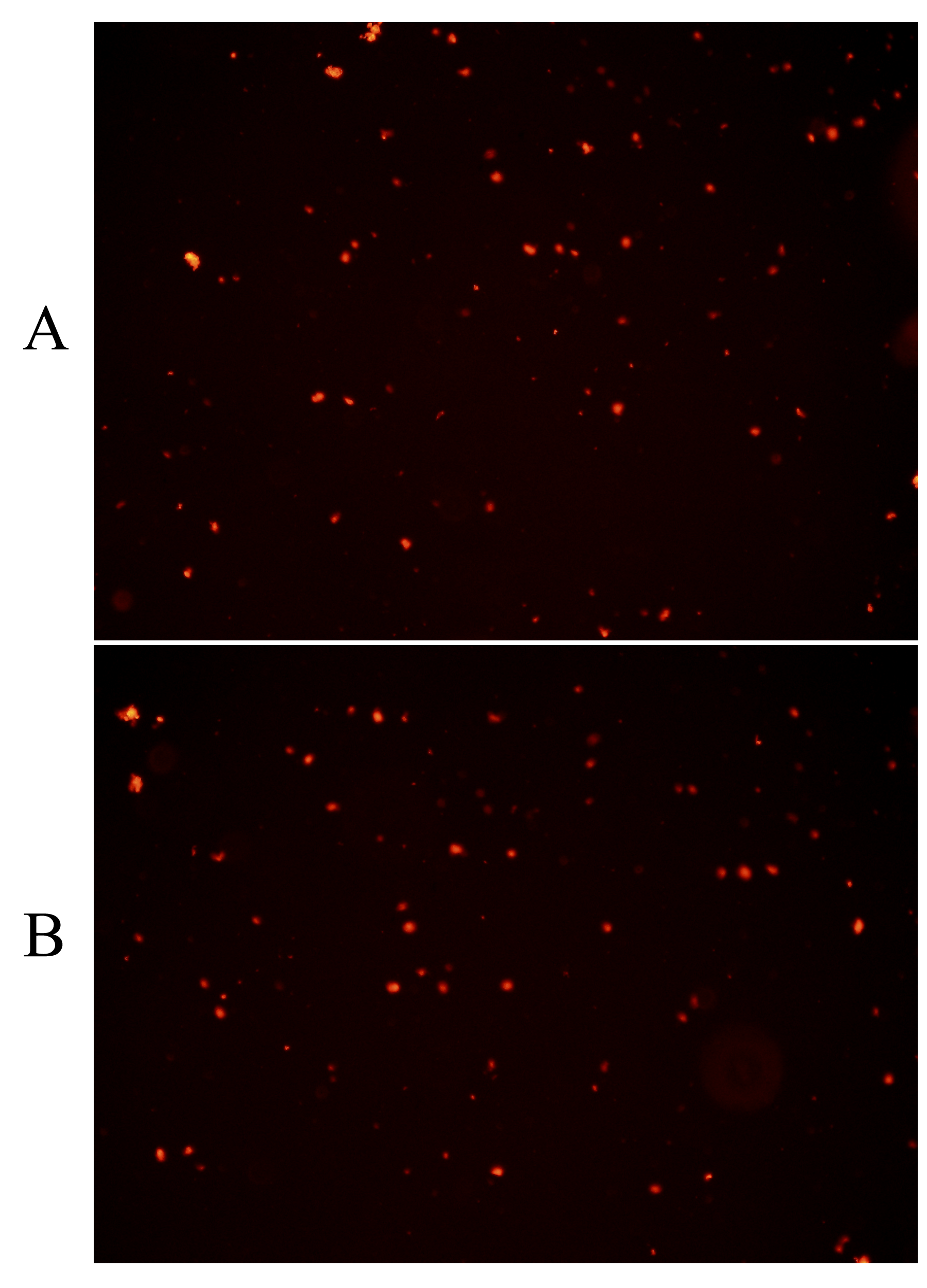
**

**Figure S1. Identification of PAVECs by uptake of acetylated low density lipoproteins (Ac-LDL).** The uptake of Ac-LDL was determined by incubating PAVECs with 10 μg/ml of 1,1’-dioctadecyl-3,3,3’,3’-tetramethylindocarbocyanine (DiI)-labelled Ac-LDL (Invitrogen, USA) in cell medium for 12 h at 37 °C. Then the cells were washed three times with PBS, detached by trypsinization and analyzed by fluorescence microscopy. A: Primary PAVECs. B: The third generation PAVECs.
